# Multiple Country and Breed Genomic Prediction of Tick Resistance in Beef Cattle

**DOI:** 10.3389/fimmu.2021.620847

**Published:** 2021-06-23

**Authors:** Fernando Flores Cardoso, Oswald Matika, Appolinaire Djikeng, Ntanganedzeni Mapholi, Heather M. Burrow, Marcos Jun Iti Yokoo, Gabriel Soares Campos, Claudia Cristina Gulias-Gomes, Valentina Riggio, Ricardo Pong-Wong, Bailey Engle, Laercio Porto-Neto, Azwihangwisi Maiwashe, Ben J. Hayes

**Affiliations:** ^1^ Embrapa Pecuária Sul, Bagé, Brazil; ^2^ The Roslin Institute and R(D)SVS, University of Edinburgh, Edinburgh, United Kingdom; ^3^ Centre for Tropical Livestock Genetics and Health (CTLGH), Roslin Institute, University of Edinburgh, Edinburgh, United Kingdom; ^4^ Department of Agriculture and Animal Health, University of South Africa, Florida, South Africa; ^5^ Faculty of Science, Agriculture, Business and Law, University of New England, Armidale, NSW, Australia; ^6^ Queensland Alliance for Agriculture and Food Innovation, University of Queensland, St Lucia, QLD, Australia; ^7^ Commonwealth Scientific and Industrial Research Organisation (CSIRO) Agriculture and Food, St Lucia, QLD, Australia; ^8^ Animal Production Unit, Agricultural Research Council, Irene, South Africa

**Keywords:** beef cattle, genomic selection, ticks, tropical adaptation, host resistance

## Abstract

Ticks cause substantial production losses for beef and dairy cattle. Cattle resistance to ticks is one of the most important factors affecting tick control, but largely neglected due to the challenge of phenotyping. In this study, we evaluate the pooling of tick resistance phenotyped reference populations from multi-country beef cattle breeds to assess the possibility of improving host resistance through multi-trait genomic selection. Data consisted of tick counts or scores assessing the number of female ticks at least 4.5 mm length and derived from seven populations, with breed, country, number of records and genotyped/phenotyped animals being respectively: Angus (AN), Brazil, 2,263, 921/1,156, Hereford (HH), Brazil, 6,615, 1,910/2,802, Brangus (BN), Brazil, 2,441, 851/851, Braford (BO), Brazil, 9,523, 3,062/4,095, Tropical Composite (TC), Australia, 229, 229/229, Brahman (BR), Australia, 675, 675/675, and Nguni (NG), South Africa, 490, 490/490. All populations were genotyped using medium density Illumina SNP BeadChips and imputed to a common high-density panel of 332,468 markers. The mean linkage disequilibrium (LD) between adjacent SNPs varied from 0.24 to 0.37 across populations and so was sufficient to allow genomic breeding values (GEBV) prediction. Correlations of LD phase between breeds were higher between composites and their founder breeds (0.81 to 0.95) and lower between NG and the other breeds (0.27 and 0.35). There was wide range of estimated heritability (0.05 and 0.42) and genetic correlation (-0.01 and 0.87) for tick resistance across the studied populations, with the largest genetic correlation observed between BN and BO. Predictive ability was improved under the old-young validation for three of the seven populations using a multi-trait approach compared to a single trait within-population prediction, while whole and partial data GEBV correlations increased in all cases, with relative improvements ranging from 3% for BO to 64% for TC. Moreover, the multi-trait analysis was useful to correct typical over-dispersion of the GEBV. Results from this study indicate that a joint genomic evaluation of AN, HH, BN, BO and BR can be readily implemented to improve tick resistance of these populations using selection on GEBV. For NG and TC additional phenotyping will be required to obtain accurate GEBV.

## Introduction

Ticks and tick-borne diseases are among the most important causes of production losses for beef and dairy cattle. Recent estimates of those losses range from US$22 to 30 billion per year (1). Cattle host resistance to ticks is one of the most important factors affecting the economics of tick control, with host resistance being moderately to highly heritable and representing a permanent solution requiring no extra labor or resources (2). However, breeding for host resistance is largely neglected in tick control programs due to the challenge of phenotyping for this trait and costs associated with identifying individual animal variation in resistance.

Genomic selection is typically suggested as a solution for improvement of traits that are hard or costly to measure. However, in the case of tick resistance, the trait is so labor intensive and expensive to measure that only small reference populations have been recorded in countries where ticks prevail (3–5). Therefore, for most cases pooling reference populations across breeds and countries may be the only effective way to achieve genomic estimated breeding values (GEBV) with sufficient accuracy to be useful. Pooling reference populations across countries has previously been demonstrated to improve accuracy for traits such as dry matter intake (6). In that study, differences in trait measurement were accounted for by treating dry matter intake as different, but potentially correlated traits between countries. Most studies pooling trait and genotype data across countries have attempted to do so only where the same breed of cattle is considered. For tropical beef cattle, this is difficult and would restrict the size of the reference population greatly, as so many different breeds, crossbreds and composites are used across the different countries.

In this study, we pool tick resistance phenotyped reference populations from beef cattle breeds in Australia, Brazil, and South Africa. Firstly an assessment is made of the extent of phase of linkage disequilibrium shared between the breeds, as a predictor of how much information might be transferred from breed to breed in genomic predictions when a high density SNP array is used [e.g. (7, 8)]. We then jointly analyze existing tick infestation datasets to assess the possibility of improving host resistance in cattle through multi-population, multi-trait genomic selection.

## Materials and Methods

### Phenotype, Genotype and Pedigree Data

#### Cattle Populations and Tick Data

Tick datasets were obtained from seven different cattle populations generated in Brazil, Australia and South Africa (**Table 1**). Tick species infesting cattle in Brazil and Australia are from the same genus (*Rhipicephalus microplus* and *R. australis*), whereas cattle in South Africa are additionally infested with the multi-host tick species *Amblyomma hebraeum* and *Hyalomma rufipes* and *H. truncatum.* Tick counts in South Africa were obtained from the *Rhipicephalus* (53%), *Amblyomma* (42%) and *Hyalomma* (5%) species.

**Table 1:** Tick resistance data according to population.

Population	Country of origin	Phenotype available	Number of observations	Mean ± S.D.	Min	Max	Number of genotyped/phenotyped animals^1^	Number of animals in validation set
Angus (AN)	Brazil	Log_10_ tick counts	2,263	1.54 ± 0.46	0.00	2.49	921/1,156	344
Hereford (HH)	Brazil	Log_10_ tick counts	6,615	1.47 ± 0.50	0.00	2.78	1,910/2,802	684
Brangus (BN)	Brazil	Log_e_ tick counts	2,441	4.32 ± 1.20	1.00	7.69	851/851	300
Braford (BO)	Brazil	Log_10_ tick counts	9,523	1.32 ± 0.43	0.00	2.72	3,062/4,095	1,267
Trop.Comp. (TC)	Australia	Tick scores	229	2.52 ± 0.93	0.00	5.00	229/229	74
Brahman (BR)	Australia	Tick scores	675	0.67 ± 0.74	0.00	4.00	675/675	216
Nguni (NG)	South Africa	Averaged log_e_ tick counts^2^	490	0.50 ± 0.17	0.02	0.95	490/490	157

^1^All genotyped animals had phenotype. ^2^Animal average solution from log transformed Tick counts.

Brazilian data consisted of log-transformed tick counts. Measurements were performed on occasions when large phenotypic variation existed in tick numbers, by manually counting adult female ticks that were at least 4.5 mm length on one whole side of the animal’s body (9). One to three subsequent tick counts on one side of each animal were obtained from Angus (AN) cattle between 2012 and 2017 from five different herds associated with the Promebo Breeding Program; from 9 Hereford (HH) and 10 Braford (BO) cattle herds between 2010 and 2018 in the Delta G Breeding Program; and from the Embrapa South Livestock Brangus (BN) experimental herd between 2013 and 2018.

For South African Nguni (NG) cattle, adult ticks were counted from the perineum body part under natural grazing for a continuous period of two years (2012 to 2014) from 490 Nguni animals. At least 23 tick counts were conducted for each animal throughout a two-year period, meaning at times there was little phenotypic variation for tick counts across animals. Tick counts (x) were log transformed using log10 (x + 1) to approximate normality. Data available for NG cattle was summarized as the average animal tick effect obtained in ASREML (10) after accounting for the following fixed effects: farm, month, year, sex, interaction between farm and month, and age, fitted as a covariate.

The Australian Brahman (BR) and Tropical Composite (TC) animals had estimates of tick counts derived from tick scores. Tick scores of adult female ticks that were > 4.5 mm in diameter on the left side of each animal, were on a 0 - 5 scale where 0 was no ticks, 1 was ≤ 10 ticks, 2 was 11 - 30 ticks, 3 was 31 - 80 ticks, 4 was 81 - 150 ticks, and 5 > 150 ticks. Tick scores are less accurate and less informative than tick counts, however there is a high genetic correlation between the two (11). Statistics of the different datasets (numbers, means and distributions) are also presented in **Table 1**.

#### Genotypes and Pedigree

Pedigree information were available and used in the analyses of the Brazilian populations only. All populations were genotyped using the Illumina SNP BeadChip technology (Illumina Inc., San Diego, CA, USA) with marker densities varying from 27k to 150k given by commercially available chips. Genotype quality control (QC) was implemented for all populations. In the case of Brazilian data QC was performed by R/SNPStats package (12). Samples with genotyping rate (call rate - CR) < 0.90, heterozygosity rate – calculated as the proportion of heterozygote genotypes within all autosomal markers of an animal – with 3 SD above or below the observed population mean, mismatching sex, and duplicate records were removed. These per animal QC criteria were applied to assure sample DNA high quality, lack of contamination or misidentification. Only SNPs mapped to autosomes with CR > 0.98, minor allele frequencies (MAF) > 0.03, and not in highly significant deviation Hardy–Weinberg equilibrium (P > 10^−7^) were considered in the analyses. In addition, only the SNP with highest MAF was retained when SNPs were observed in the exact same position or the genotypes were highly correlated (r > 0.98). Similar quality control steps were applied to the Australian populations and NG, with the addition that genotype calls with a GC score below 0.6 were set to missing and were filled in with imputation using FImpute (13). After quality control, genotypes from all populations were imputed to a common high-density panel of 332,468 markers distributed throughout the 29 bovine autosomal chromosomes. Brangus, Braford and Hereford populations were imputed using the FImpute software (13) and an HD sample of 340 animals available at Embrapa datasets for these breeds. Angus, Brahman, Nguni and Tropical Composites were imputed using the 1,000 bull genome project reference, which includes 305, 122, 0 and 30 sequences respectively from those imputed breeds and 2,603 in total from 107 breeds (13), and findhap software (14).

### Population Genomic Parameters

#### Linkage Disequilibrium

Linkage disequilibrium (LD) was estimated for each chromosome between adjacent pairs of SNPs as the squared correlation statistic (r^2^) (15), which can be calculated as follows:

(1) $$r^{2}=\frac{{\left(\rho_{AB}\rho_{ab}-\rho_{Ab}\rho_{aB}\right)}^{2}\;}{\left(\rho_{A}\rho_{a}\rho_{B}\rho_{b}\right)}$$

where *ρ_A_*, *ρ_a_*, *ρ_B_* and *ρ_b_* are the frequencies of alleles A, a, B and b, respectively; *ρ_AB_*, *ρ_ab_*, *ρ_Ab_* and *ρ_aB_* are the haplotype frequencies among alleles in the population.

#### Persistence of Phase Across Breeds

To investigate the LD phase between two specific breeds, the Pearson correlation *r_ij(A)_* and *r_ij(B)_* for a common set of adjacent SNPs between populations A and B was calculated using the following equation (16):

(2) $$R_{A,B}=\;\frac{\Sigma_{(i,j)\in l}\left(r_{ij(A)}-{\bar{r}}_{A}\right)\left(r_{ij(B)}-{\bar{r}}_{B}\right)}{S_{A}S_{B}}$$

where *R_A,B_* is the correlation of phase between *r_ij(A)_* in population A and *r_ij(B)_* in population B, *S_A_* and *S_B_* are the standard deviation of *r_ij(A)_* and *r_ij(B)_* respectively, and ${\bar{r}}_{A}$ and ${\bar{r}}_{B}$ are the average *r_ij_* across adjacent SNP i and j within the interval l for populations A and B for a common set of markers. The *r^2^* and *r* values were estimated using adjacent SNPs with the ld_estimate R scripts (16).

#### Allele Frequencies and Principal Components

Additionally, Pearson correlations were calculated between allele frequencies of all populations across the 332,468 SNP markers used in the present study and a principal components analysis (PCA) plot of all animals by breed was obtained from genotype data using PreGSf90 software (17).

### Statistical Models and Analysis

#### Multivariate Genomic BLUP

Data quality checks for the Brazilian populations were performed using R program (18). Contemporary groups (CG) were formed by animals from the same farm, sex, year and season of birth, sex and management group and date of tick count evaluations. Contemporary groups with less than five animals and data exceeding 3.5 SD above or below the mean of the CG were excluded.

The statistical models for all populations except NG included the fixed effect of contemporary groups; the linear covariate effects of individual zebu breed composition and heterozygosity, according to their expected values based on pedigree information, and the linear and quadratic covariate effects of animal age. For pre-adjusted NG data only an overall mean was fitted as fixed effect. Additionally, direct additive genetic, permanent environmental and residual random effects were included for the Brazilian populations that had repeated tick count measures and only the direct additive genetic and residual effects were considered for Australian and South African populations with single measurements. The models can be represented in matrix notation by the following equations:

(3) $$\begin{array}{c} \left[\begin{array}{c} y_{AN}\\ y_{HH}\\ y_{BN}\\ y_{BO}\\ y_{TC}\\ y_{BR}\\ y_{NG} \end{array}\right]=\left[\begin{array}{cccc} X_{AN} & 0 & \cdots & 0\\ 0 & X_{HH} & \ldots & 0\\ \ldots & \ldots & \ddots & \ldots \\ 0 & 0 & \cdots & X_{NG} \end{array}\right]\left[\begin{array}{c} \beta_{AN}\\ \beta_{HH}\\ \beta_{BN}\\ \beta_{BO}\\ \beta_{TC}\\ \beta_{BR}\\ \beta_{NG} \end{array}\right]+\left[\begin{array}{cccc} Z_{AN} & 0 & \cdots & 0\\ 0 & Z_{HH} & \ldots & 0\\ \ldots & \ldots & \ddots & \ldots \\ 0 & 0 & \cdots & Z_{NG} \end{array}\right]\left[\begin{array}{c} u_{AN}\\ u_{HH}\\ u_{BN}\\ u_{BO}\\ u_{TC}\\ u_{BR}\\ u_{NG} \end{array}\right]\\ +\left[\begin{array}{cccc} W_{AN} & 0 & \cdots & 0\\ 0 & W_{HH} & \ldots & 0\\ \ldots & \ldots & \ddots & \ldots \\ 0 & 0 & \cdots & 0 \end{array}\right]\left[\begin{array}{c} p_{AN}\\ p_{HH}\\ p_{BN}\\ p_{BO}\\ 0\\ 0\\ 0 \end{array}\right]+\left[\begin{array}{c} e_{AN}\\ e_{HH}\\ e_{BN}\\ e_{BO}\\ e_{TC}\\ e_{BR}\\ e_{NG} \end{array}\right], \end{array}$$

where: the **y** *_b_*’s are vectors of the tick infestation trait for each *b*th breed, b=AN, HH, BN, BO, TC, BR, and NG, respectively for Angus, Hereford, Brangus, Braford, Tropical Composite, Brahman and Nguni breeds. Similarly, for each *b*th breed, **β** *_b_*’s are the vectors of systematic effects, **u** *_b_*’s are the vectors of random direct additive genetic effects, **p** *_b_*’s are the vector of random permanent environmental effects (only pertaining to AN, HH, BN and BO that have repeated measures), and the **e** *_b_*’s are the corresponding vectors of random residual effects. Additionally, each *b*th breed also has its own incidence matrices of systematic, direct additive genetic, and animal permanent environmental effects, respectively represented by **X** *_b_*’s, **Z** *_b_*’s, and **W** *_b_*’s.

As Brazilian populations had ungenotyped individuals with phenotype, we used a multi-trait single step genomic BLUP (ssGBLUP) approach (19, 20), with the following assumptions about the prior distributions of the model random parameters:

(4) $$\left[\begin{array}{c} u_{AN}\\ u_{HH}\\ u_{BN}\\ u_{BO}\\ u_{TC}\\ u_{BR}\\ u_{NG} \end{array}\right]\sim N\left(\begin{array}{c} \left[\begin{array}{c} 0\\ 0\\ 0\\ 0\\ 0\\ 0\\ 0 \end{array}\right] \end{array},\left[\begin{array}{lllllll} \sigma_{u_{AN}}^{2} & \sigma_{u_{AN,HH}} & \sigma_{u_{AN,BN}} & \sigma_{u_{AN,BO}} & \sigma_{u_{AN,TC}} & \sigma_{u_{AN,BR}} & \sigma_{u_{AN,NG}}\\ & \sigma_{u_{HH}}^{2} & \sigma_{u_{HH,BN}} & \sigma_{u_{HH,BO}} & \sigma_{u_{HH,TC}} & \sigma_{u_{HH,BR}} & \sigma_{u_{HH,NG}}\\ & & \sigma_{u_{BN}}^{2} & \sigma_{u_{BN,BO}} & \sigma_{u_{BN,TC}} & \sigma_{u_{BN,BR}} & \sigma_{u_{BN,NG}}\\ & & & \sigma_{u_{BO}}^{2} & \sigma_{u_{BO,TC}} & \sigma_{u_{BO,BR}} & \sigma_{u_{BO,NG}}\\ & & & & \sigma_{u_{TC}}^{2} & \sigma_{u_{TC,BR}} & \sigma_{u_{TC,NG}}\\ & & Symm. & & & \sigma_{u_{BR}}^{2} & \sigma_{u_{BR,NG}}\\ & & & & & & \sigma_{u_{NG}}^{2} \end{array}\right]⊗H\right),$$

where $\sigma_{u_{b}}^{2}$ is the additive genetic variance of the *b*th breed, $\sigma_{u_{b,c}}$ the additive genetic covariance between the *b*th and *c*th breeds, ⊗ denotes the direct product between the matrices, and **H** is a relationship matrix constructed by combining the pedigree and genomic relationship matrices (20–22). Although **H** is complex (22), its inverse, which is needed in the computations, has the simpler form (19):

(5) $$H^{-1}=A^{-1}+\left[\begin{array}{cc} 0 & 0\\ 0 & {\left(0.95G+0.05A_{22}\right)}^{-1}\;-\;A_{22}^{-1} \end{array}\right]$$

Here **G** is the genomic relationship matrix constructed as shown in the first method proposed by VanRaden (23) using current allele frequencies averaged across breeds. While theoretically correct for multiple breed populations, adjusting for breed specific allele frequencies was not performed because it has been shown to have negligible impact on prediction accuracy (24). Moreover, **A** ^-1^ is the inverse of the numerator relationship matrix and *A_22_* is the numerator relationship matrix for genotyped animals only. Since, there were no genetic ties between populations through pedigree, all the relationship between populations was genomic and given by **G**.

Furthermore,

(6) $$\left[\begin{array}{c} p_{AN}\\ p_{HH}\\ p_{BN}\\ p_{BO} \end{array}\right]\sim N\left(\begin{array}{c} \left[\begin{array}{c} 0\\ 0\\ 0\\ 0 \end{array}\right] \end{array},\;\left[\begin{array}{cccc} \sigma_{p_{AN}}^{2} & 0 & 0 & 0\\ & \sigma_{p_{HH}}^{2} & 0 & 0\\ & & \sigma_{p_{BN}}^{2} & 0\\ Symm. & & & \sigma_{p_{BO}}^{2} \end{array}\right]⊗I\right)$$

and

(7) $$\left[\begin{array}{c} e_{AN}\\ e_{HH}\\ e_{BN}\\ e_{BO}\\ e_{TC}\\ e_{BR}\\ e_{NG} \end{array}\right]\sim N\left(\begin{array}{c} \left[\begin{array}{c} 0\\ 0\\ 0\\ 0\\ 0\\ 0\\ 0 \end{array}\right] \end{array},\left[\begin{array}{ccccccc} \sigma_{e_{AN}}^{2} & 0 & 0 & 0 & 0 & 0 & 0\\ & \sigma_{e_{HH}}^{2} & 0 & 0 & 0 & 0 & 0\\ & & \sigma_{e_{BN}}^{2} & 0 & 0 & 0 & 0\\ & & & \sigma_{e_{BO}}^{2} & 0 & 0 & 0\\ & & & & \sigma_{e_{TC}}^{2} & 0 & 0\\ & Symm. & & & & \sigma_{e_{BR}}^{2} & 0\\ & & & & & & \sigma_{e_{NG}}^{2} \end{array}\right]⊗I\right),$$

where $\sigma_{p_{b}}^{2}$ and $\sigma_{e_{b}}^{2}$ are respectively the permanent environmental and residual variances of the *b*th breed, and **I** represents an identity matrix. These permanent environmental and residual effects were necessarily uncorrelated between traits due to the mutually exclusive assignment of individuals to breeds.

The (co)variance components and genetic parameters were estimated using Bayesian inference by Gibbs sampling, with the Gibbs2f90 program (25) in multi-trait analysis and using a linear animal model, considering the phenotypic measurement of tick infestation in each population as a different trait that is potentially genetically correlated among populations. Analyses consisted of a single chain of 1,000,000 cycles, with a burn-in period of 100,000 cycles and a thinning interval of 50 cycles. The posterior estimates were obtained using the Postgibbsf90 program (25) and the R/coda package (26). These estimated (co)variance components were used to obtain best linear unbiased predictions (BLUP) of tick resistance breeding values under multi- and single-trait scenarios using the Blupf90 software (25).

#### Univariate Genomic BLUP

Univariate breed-specific analyses were performed considering the records and the marginal model for each *b*th breed derived from multi-trait model described above (Equation [3]), as follows:

(8) $$y_{b}=X_{b}\beta_{b}+\;Z_{b}u_{b}+\;W_{b}p_{b}+e_{b}.$$

Similarly, the marginal distributional assumptions were derived from equations [4], [6], and [7] as:

(9) $$u_{b}\sim N\left(0,H\sigma_{ub}^{2}\right),p_{b}\sim N\left(0,I\sigma_{p_{b}}^{2}\right),\;and\;e_{b}\sim N\left(0,I\sigma_{eb}^{2}\right).$$

These single trait/breed analyses were used as controls to check the advantages of jointly analyzing all breeds, and they used the same variance component estimates as the multi-trait analyses to maintain equivalent dispersion of breeding values for each breed under both strategies (single and multi population predictions).

#### Validation of Genomic Predictions

The utility of our reference populations to predict tick resistance and future phenotypes in single and multiple trait/breed genomic analyses was evaluated using the linear regression (LR) approach proposed by Legarra and Reverter (27). This method measures the correlation of estimated breeding values (*û*) between whole (*w*) and partial (*p*) datasets between subsequent genetic evaluations when phenotypes are added for validation animals,

$$\rho_{w,p}=\;\frac{cov\left({\hat{u}}_{w,}{\hat{u}}_{p}\right)}{\sqrt{var\left({\hat{u}}_{w}\right)var\left({\hat{u}}_{p}\right)}},$$

which is a function of the prediction accuracy with expected value of *E(ρ*_w,p_)≈ *acc_p_*/*acc_w_*. Here *acc* is the “population accuracy”, i.e. the correlation between true and estimated breeding values in the candidates for selection, which is a property of a population, not of an individual (27). Here, the whole dataset *w* included the combined set of all genotyped and phenotyped animals for all breeds (ranging from 229 to 3,062 individuals) in the multivariate analyses and the full set of genotyped and phenotyped animals for each breeds for univariate analyses. The partial datasets were derived for each population by two strategies, the first was the old-young where only 2/3 of the phenotypes pertaining to the older animals were retained in the partial data and the remaining 1/3 younger animals had their phenotypes set to missing and served as the validation group in both, uni and multivariate analyses. Additionally, for multivariate analyses only, a second strategy referred as other-pops consisted of removing from the analysis all phenotypes of the target population for validation and deriving predictions exclusively from the genetic correlations of the target with the other populations/breeds with full datasets included. When the *P_w,p_* is large (closer to one), the partial data reliably predicts the whole data. As additional validation statistics, we calculated the predictive ability defined as the correlation between phenotypes adjusted for fixed and permanent environmental effects (**y** ^*^ **=y** *_b_*-**X** *_b_*β*_b_*-**W** *_b_* **p** *_b_*) and ***û*** *_p_* (*r*(y^*^,û*_p_*)) (28), where **û** *_p_* is the GEBV with partial data; and the slope of the regression of **û** *_w_* on **û** *_p_*(*β_w,p_*), which was used to evaluate the degree of inflation/deflation of the genomic predictions.

## Results and Discussion

### Population Genomic Structure and Diversity

#### Genomic Diversity

**Figure 1:**
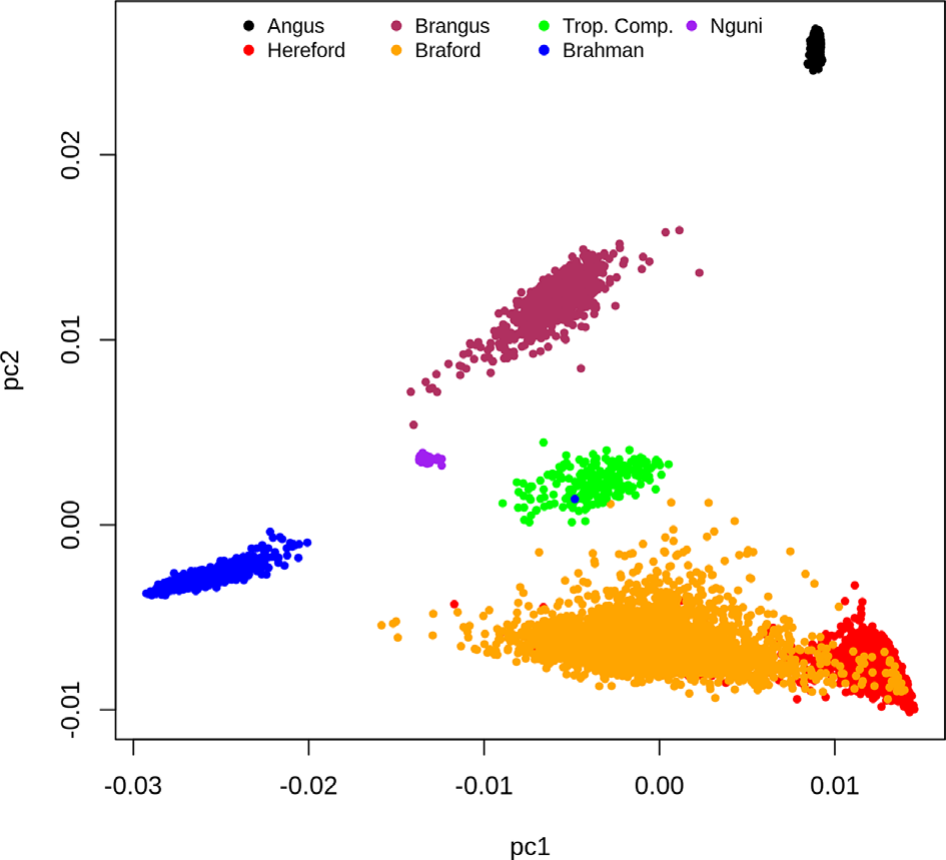
Dispersion of individuals according to the first and second principal components of the **G** matrix, colored by breed.

Based on the dispersion of individuals according to the first and second principal components (PC) of the **G** matrix (**Figure 1**), it is possible to identify the distinct genotypic constitution of the breeds included in the present study and the magnitude of genetic distance among them. If we analyze this PCA plot (**Figure 1**) from a perspective of a triangular form, we would place BR, HH and AN animals at the vertexes, respectively located at the lower left, the lower right and upper right regions of the plot. The composites BO and BN animals are respectively scattered on the lower and upper sides of the triangle that connect their founder breeds vertexes. Therefore the first PC mostly discriminates the percentage of indicine origin while the second PC genetically distinguished the AN and HH origin. The TC animals that are an admixture of Brahman, Sanga (represented mainly by Afrikaner but also some Tuli) and British/European (primarily Shorthorn and Hereford with some Charolais) breeds were scattered at the center of the triangle. Finally, the NG that is also part of the Sanga breed grouping fell in the PCA plot relatively close to TC samples towards the center upper left of our perspective triangle.

The clusters for the NG and AN samples have low dispersion reflecting their genetic homogeneity as opposed to more scattered and therefore heterogeneous samples of composite breeds (BN, BO, BR and TC) and HH (**Figure 1**). However, this could also reflect some ascertainment bias in the SNP on the Bovine HD array. The BO was the most genetically diverse breed group and had partial overlap with the HH samples. This was not surprising because the Delta G population from which records were derived for the present study is a joint Breeding Program for purebred (as opposed to full blood) Herefords and Brafords that range from 1/16 to 7/8 of zebu proportion (29).

#### Linkage Disequilibrium

**Figure 2:**
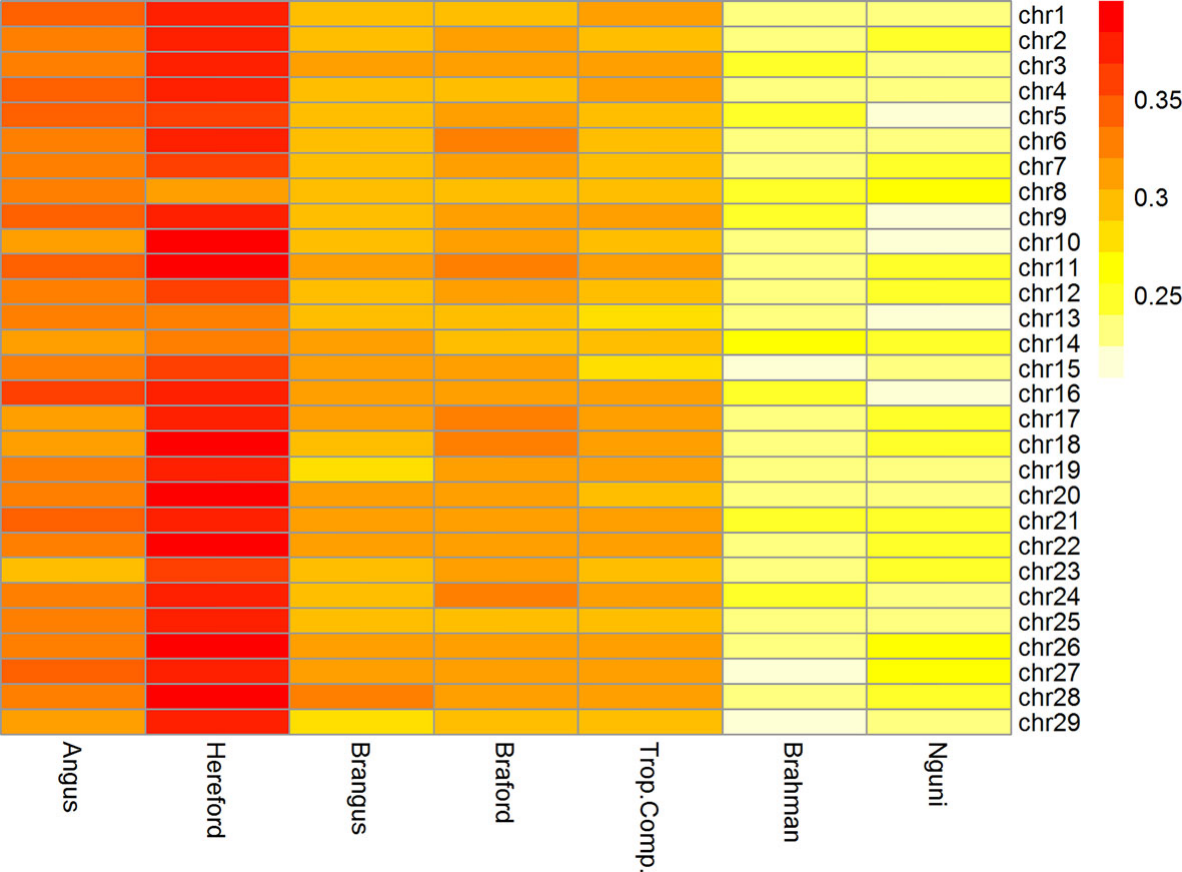
Heatmap of linkage disequilibrium (*r^2^*) between adjacent markers of the 332k SNP panel by breed and chromosome.

The mean ± standard deviation *r* ^2^ between adjacent SNPs ranged from 0.24 ± 0.34 to 0.37 ± 0.35 across all chromosomes for cattle populations from Brazil, Australia, and South Africa. The *r* ^2^ among chromosomes was similar within all breeds as observed in **Figure 2**.

The Brazilian populations of British origin, AN (0.33 ± 0.35) and HH (0.37 ± 0.35), had higher LD values than the other populations. The composite breeds from Brazil, BN (0.30 ± 0.28) and BO (0.31 ± 0.27) and TC (0.30 ± 0.29) from Australia had intermediate *r* ^2^ values. Conversely, the NG (0.24 ± 0.34) and BR (0.24 ± 0.27) breeds had lower *r* ^2^ among the studied breeds. Lower LD estimates at short distances are an indication of large ancestral population sizes and have been reported for indicine cattle compared to taurine cattle (30–32). This is consistent with LD estimates in the present study and in the case of NG, an African taurine population of the Sanga group, a previous report has also found lower short distance LD compared to European taurine cattle (33).

Furthermore, the *r* ^2^ was > 0.3 for more than 40% of neighboring SNPs only in HH and BO breeds (data not shown). In relation to the other breeds, the mean *r* ^2^ > 0.3 were about 30% for NG and BR and, around 38% for BN, AN, and TC.

Genomic selection relies on LD between QTLs and flanking SNPs and simulation results demonstrated that, to obtain sufficiently accurate GEBVs to be useful for breeding decisions, an average *r^2^* between adjacent markers of 0.20 would suffice [e.g. (34)]. This was achieved for all chromosomes within all studied breeds with our 332k SNP panel (**Figure 2**).

#### Persistence of Phase Across Breeds

**Figure 3:**
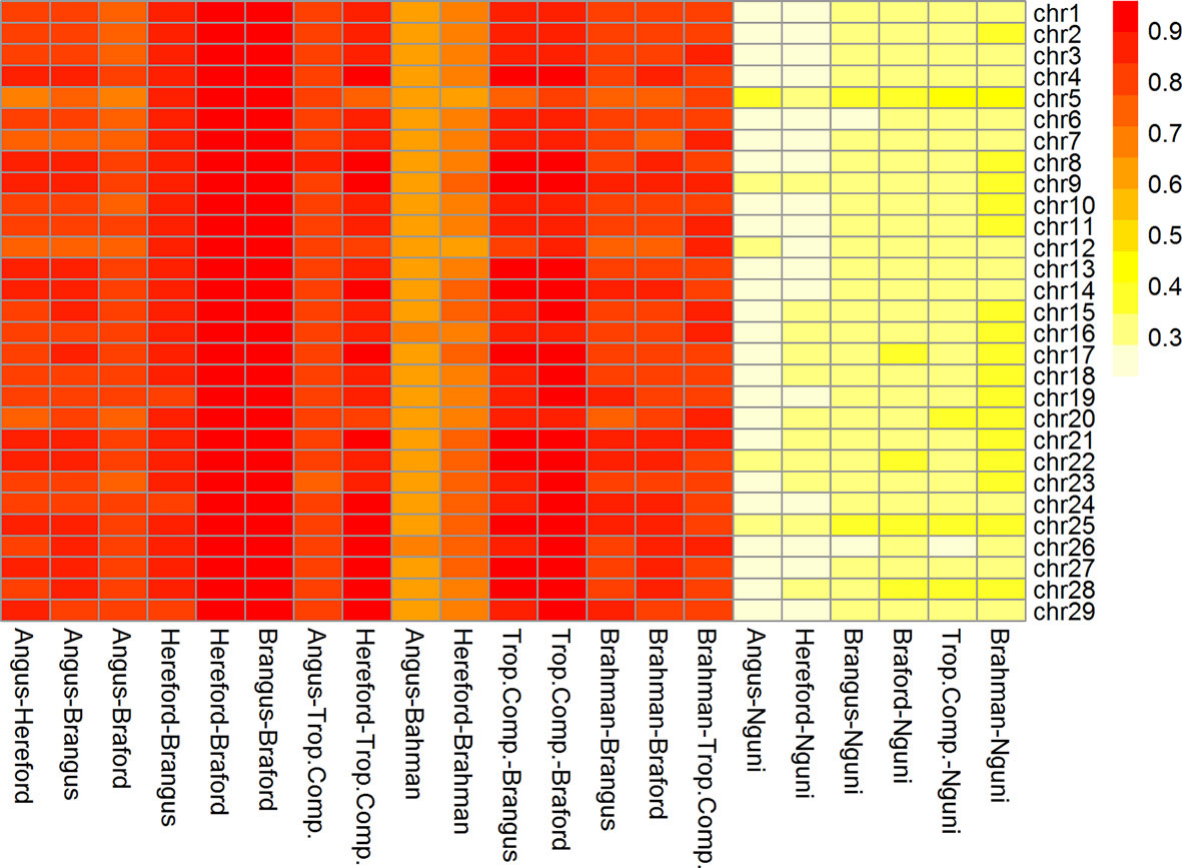
Heatmap of correlation of phase between adjacent markers among breeds and chromosomes (332k panel by chromosome).

The correlations (R*_A,B_*) of linkage phase were used to estimate the haplotype-sharing between pairs of adjacent SNPs across breeds (**Table 2** and **Figure 3**). The R*_A,B_* statistic is useful because the accuracy of genomic selection across breeds relies on persistence of the LD phase, though not actually between pairs of SNPs but between SNP and QTL (7, 8). If the correlation between pairs of adjacent SNPs is high, then the correlation between the QTL and SNP should be high as well. In general, if two populations have a high positive *R_A,B_* value, it suggests high LD and the same haplotype phase in both populations; however, a high negative value indicates high LD but with reverse linkage phase (7).

**Table 2:** Average persistence of phase for adjacent markers (above the diagonal) and correlation of allele frequencies (below the diagonal) between different populations.

Population	Angus	Hereford	Brangus	Braford	Tropical Composite	Brahman	Nguni
Angus		0.81	0.81	0.77	0.81	0.63	0.27
Hereford	0.72		0.87	0.95	0.87	0.69	0.28
Brangus	0.77	0.60		0.92	0.88	0.82	0.31
Braford	0.69	0.88	0.77		0.89	0.81	0.32
Tropical Composite	0.67	0.69	0.76	0.81		0.83	0.32
Brahman	0.21	0.15	0.60	0.54	0.55		0.35
Nguni	0.48	0.43	0.66	0.64	0.67	0.67	

The *R_A,B_* correlation between adjacent SNP pairs across chromosomes among Brazilian populations ranged from 0.77 (AN *vs*. BO) to 0.95 (HH *vs*. BO) (**Table 2**). The correspondence of linkage phase among the Brazilian composites (BN and BO) and Australian populations (BR and TC) was on average 0.80 and the highest *R_A,B_* value was 0.89 between TC *vs*. BO (**Table 2**). Among AN, HH and Australian populations, the *R_A,B_* values varied from 0.63 (AN *vs*. BR) to 0.87 (HH *vs*. TC). The smallest values were found for NG vs. all other breeds (**Figure 3**). As observed in the LD (**Figure 2**), the average *R_A,B_* values also vary across chromosomes within population pairs (**Figure 3**). This information is useful to choose marker density that should be determined according to the lower bound of the chromosome *R_A,B_* averages, particularly if those chromosomes harbor mutations potentially associated with traits of interest.

De Roos et al. (7) pointed out that finding markers in LD with QTL across divergent breeds, such as Australian Angus and New Zealand Jersey, would require a panel of approximately 300,000 markers. This is aligned with our choice of marker density (332k), but even so, *R_A,B_* was relatively low for several breed pairs, especially those including the NG breed, and between taurine AN and HH and indicine BR (**Table 2**), which are the most divergent breed groups. The highest *R_A,B_* value found between HH and BO indicates the highest proportion of SNP sharing the same linkage phase for these breeds and was in agreement with previous findings within the same populations and a 50k panel (32). It is important to point out however that genomic prediction across-population or across-breed accuracies rely not only on the persistence of LD phase across populations, but also on the trait genetic architecture and size of the reference populations (35).

#### Allele Frequency Correlations

Differences across populations were seen in terms of allele frequencies (**Table 2**) with correlations between populations ranging from 0.15 (HH and BR) to 0.88 (HH and BO). Composite breeds had high correlations among themselves and with their taurine founder breeds (AN with BN and HH with BO). As expected low correlations were found between the indicine BR and the taurine AN and HH, moderately high correlations were observed between BR and composites (BN, BO and TC) and medium to moderately high correlations of NG with all the other breeds.

### Genomic Selection Parameters

#### Genetic Correlations and Heritabilities

All 39 estimated variance components passed a convergence test based on the Geweke’s criterion (36). The mean ± standard deviation effective number of independent samples (37) for the genetic parameters was 402 ± 857, ranging from 46 to 4,594. This wide range of values reflects distinct data information content to estimate the posterior means of genetic correlations and heritabilities across the different populations (**Table 3**). Hereford showed the lowest *h^2^* for tick counts among the studied breeds, while the other Brazilian commercial populations had low to moderate values, in line with heritabilities typically found for this trait (5). The Australian BR and TC scores and South African NG averaged counts had larger estimated values around 0.40 in our multi-trait analysis, and were are considerably higher than previous results obtained within-population under single trait analyses of 0.15 for BR (11). Nonetheless, a similarly high *h* ^2^ value of 0.42 was reported for another Tropical Composite Australian population, the Belmont Red (38) and depending on the time of the year *h* ^2^ for perineum tick counts ranged between 0.00 and 0.58 (39). The wide range of *h* ^2^ estimates for tick resistance in cattle found in the present and other studies is related to differences in phenotyping, environmental control and intrinsic population characteristics. It is also important to highlight that the NG trait is the average of log transformed tick count over multiple observations. This may have lowered the environmental variance and, consequently, inflated the estimated *h* ^2^ for the NG breed. The estimated repeatability for the AN, HH, BN, BO populations that had repeated measures were, respectively 0.30, 0.16, 0.37, 0.28, with corresponding standard errors (SE) of 0.001 or less.

**Table 3:** Posterior mean and time series standard errors for genetic correlations (above diagonal) and heritabilities (diagonal) of tick resistance measures across different populations.

Population	Angus	Hereford	Brangus	Braford	Tropical Composite	Brahman	Nguni
Angus	0.27 ± 0.001	0.32 ± 0.03	0.65 ± 0.001	0.42 ± 0.03	0.15 ± 0.04	0.17 ± 0.03	0.17 ± 0.03
Hereford		0.05 ± 0.001	0.39 ± 0.01	0.35 ± 0.01	0.22 ± 0.04	0.01 ± 0.03	0.05 ± 0.06
Brangus			0.21 ± 0.003	0.87 ± 0.01	0.32 ± 0.02	0.47 ± 0.01	0.29 ± 0.05
Braford				0.17 ± 0.001	0.48 ± 0.02	0.59 ± 0.01	0.14 ± 0.05
Tropical Composite					0.42 ± 0.01	0.28 ± 0.02	-0.01 ± 0.05
Brahman						0.39 ± 0.01	0.18 ± 0.03
Nguni							0.37 ± 0.02

The largest genetic correlation among all studied populations was observed between BN and BO. These two breeds are composites with about 3/8 of zebu composition, mostly Nelore in our samples, and the other 5/8 being taurine of British origin, Angus or Hereford. This result indicates a very similar additive genetic mechanism for tick resistance in both populations. The second largest relationship was observed between AN and its composite with zebu, the BN breed, and this is not surprising because the average expected contribution of AN to BN is approximately 62.5%. Even though AN and BN are more closely related than BN and BO, the higher genetic correlation between the latter pair could be related to greater indicine impact on tick resistance (5, 40, 41). Braford and BR had the third largest genetic association for tick resistance and the only other with a value above 0.5. Brangus and BR, BO and TC, and BO and HH had values around 0.4 showing some level of additive genetic association, but not strong enough to decisively contribute to the sharing of information among reference populations designed for tick resistance prediction across breeds. All other breed pairs showed weak genetic correlations between tick phenotypes, particularly for the NG breed where there was no useful association pertaining to the improvement of resistance.

#### Genomic Predictive Ability

Predictive ability, as a measure of the GEBV to predict the observed phenotype, was improved under the old-young validation for three of the seven populations using the multi-trait approach compared to a single trait within-population prediction. Moreover, we observed improvement for the partial and whole data GEBV correlation in all cases by using multi-trait analysis under old-young (**Table 4**), with relative improvements ranging from 3% for BO to 64% for TC. Moreover, the multi-trait analysis was useful to correct typical over-dispersion of GEBV in all populations except for the BO breed that had no such issue in both analyses – uni or multivariate for the old-young validation strategy (**Table 4**).

**Table 4:** Predictive ability^1^ [r(y *,*û* ***_p_***)], regression coefficient (*β_w,p_*) and correlation between genomic breeding values (**û**) predicted from whole (*w*) and partial^2^ (*p*) data using uni and multivariate ssGBLUP population analyses.

Population	*r* ***(*** *y* ^*^, *û* ***_p_)***			*β_w,p_*			*Δ_w,p_*		
	**Uni old-young**	**Multi old-young**	**Multi other-pops**	**Uni old-young**	**Multi old-young**	**Multi other-pops**	**Uni old-young**	**Multi old-young**	**Multi other-pops**
Angus	0.15	0.16	0.07	0.92	1.06	1.06	0.50	0.58	0.22
Hereford	0.05	0.05	0.04	0.99	1.01	0.65	0.56	0.58	0.40
Brangus	0.22	0.25	0.22	0.88	0.93	1.46	0.67	0.72	0.57
Braford	0.24	0.24	0.17	1.01	1.00	1.45	0.76	0.78	0.56
Tropical Composite	-0.06	0.00	0.21	0.32	0.53	1.74	0.14	0.23	0.35
Brahman	0.13	0.13	0.20	0.77	0.83	1.44	0.57	0.64	0.43
Nguni	0.04	0.04	-0.04	0.79	1.00	-2.11	0.18	0.20	-0.04

^1^Correlation between phenotypes adjusted for fixed and permanent environmental effects and û_p_.

^2^Partial datasets derived by two strategies: old-young = excluding phenotypes of 1/3 younger animals as validation group; and other-pops = removing all phenotypes of the target population for validation.

The multivariate validations based on data of other populations only (other-pops), which were included to evaluate the possibility of predicting tick resistance for populations that do not have a reference population for this trait, had in general a poorer predictive performance compared to uni and multivariate old-young validations for all parameters evaluated (**Table 4**). These results emphasize the importance of having consistent phenotyping strategies and genotypes for populations in which improving tick resistance is a goal. Nonetheless, prediction ability retained estimated values that can be considered useful for applied purposes for BO and BN, and was improved for TC and BR (**Table 4**). These results are an indication that a breed without reference population for tick resistance but with high genomic relationship and persistence of LD phase with one or more of our measured populations could be targeted for selection through such predictions. The greatest challenge for such application however is to estimate meaningful trait genetic correlation parameters with the reference populations.

Braford was the breed with highest predictive ability in all criteria in all analyses: uni or multivariate (**Table 4**). This was not surprising since BO has the largest reference population in our study and moderate trait heritability. Furthermore, the viability of implementing genomic selection for this breed has been previously demonstrated with a subset of our BO/HH data (4). Despite being highly correlated with BN and BR, there was minor improvement for BO in the old-young multi-trait analysis, as there was already considerable information for this breed. In fact BN and BR were the breeds that benefit most in terms of accuracy in the multi-breed multi-trait analyses, likely through their genetic linkage to BO tick resistance.

The HH breed with the second largest reference population in our sample had a low predictive ability (**Table 4**) in agreement with the very low *h* ^2^ for tick counts of this breed (**Table 3**). Nonetheless, the estimated correlations of GEBV for whole and partial data can be considered of medium value and useful enough to allow practical use of genomic selection to improve tick resistance of this breed. There was a minor improvement from old-young uni to multivariate analysis basically attributable to a medium genetic correlation with the BO breed (**Table 3**) and other breeds in the study. In both old-young analyses, HH predictions can be considered unbiased given the *β_w,p_* values close to 1 in **Table 4**.

Angus and BN breeds had similar population sizes close to 1,000 animals and over 2,200 records, their tick count *h^2^* were in the medium range, and in both populations we observed prediction results that ensure the possibility of improving tick resistance through genomic selection (**Table 4**). Brangus had, however, slightly better predictive abilities and GEBV correlations for uni- and multi-variate analyses than AN. The estimated genetic correlations were positive and of strong magnitude between BN and BO, AN and BN, and AN and BO, resulting in improvements of practical importance for all prediction measures in the old-young multivariate validations for these populations. These results support a joint evaluation to implement genomic selection for tick resistance. A similar strategy has been suggested for an international genetic evaluation of feed intake in dairy cattle for high-input production systems (6).

The largest improvements were observed for the TC population (**Table 4**), which had the smallest reference population with only 229 individuals. Nonetheless, the genetic correlation of their tick score phenotypes with other larger populations (**Table 3**), particularly the medium value with Brafords, was not strong enough to yield prediction with useful correlations to be immediately implemented in practical genomic selection. The results, however, indicate that perhaps even a modest additional effort of phenotyping in this population could suffice for future adoption of genomic prediction for tick resistance of Tropical Composites.

Even though the BR breed had a modest reference population of 675 animals, it had the third highest GEBV correlations in old-young uni and multivariate analyses (**Table 4**) due to the high heritability of their tick scores. With a high genetic correlation with the BO and a medium genetic correlation with BN, BR prediction accuracies and dispersions were improved when using a joint multi-population evaluation of tick phenotypes.

Finally, the NG breed had low predictive ability and GEBV correlation (**Table 4**) likely reflecting modest sample size. These results were not substantially improved when using the multivariate analysis due to overall low genetic (**Table 3**) and phase (**Table 2**) correlations of Ngunis with the other populations in our study. The poorer results and relationships for Nguni may reflect factors other than the genetic mechanisms of host tick resistance. For example there are multi-host tick species in South Africa [e.g. (3)] that are not present in either Brazil or Australia, so the counts may simply be a reflection of those different tick species possibly having different mechanisms of resistance (42).

Another factor that could also explain this lower accuracy of prediction for NG could be the time and body location of counting. In the Australian and Brazilian data, tick counts only occurred at times of the year when there was large phenotypic variation and assessing one whole side of the animal, while for the NG population perineum counts occurred throughout the year and the averaged data used in the present study included counting times that would not meet the phenotyping requirement of at least 20 ticks per side of each animal, averaged over at least 15 animals (42). Therefore, additional phenotyping and genotyping must be pursued within this breed before practical genomic selection can be implemented to increase its tick resistance.

A recent review of the scientific literature identified possibly simpler, more cost-effective phenotype(s) for tick resistance, which if developed and validated, could be used to greatly enlarge the reference populations for genomic prediction and to improve the accuracy of GEBV for this trait, as well as potentially improving tick control through cattle management (42).

Even though more extensive phenotyping should be a continuous effort to improve the accuracy of GEBV for tick resistance, old-young validation results from this study (**Tables 3**, **4**) indicate that a joint genomic evaluation of Angus, Hereford, Brangus, Braford and Brahman using multivariate genomic BLUP can be readily implemented to improve tick resistance of these populations using genomic predictions. The extent of improvement of accuracy of GEBV for a breed from the multi-population approach largely reflect the extent of LD phase between the breeds, except for cases such as BO where the reference population is already relatively large. Even for these breeds, the accuracy from using multi-breed information may be further improved if sequence data is used, provided the same mutations are segregating across the breeds (which is quite likely in composite breeds), such that correlations would be essentially 1 (43).

## Data Availability Statement

The data analyzed in this study is subject to the following licenses/restrictions: The raw datasets cannot be made available because they are the property of the Breeders and Institutions involved in generating them and this information is commercially sensitive. For scientific research purposes, the data requests should be forwarded along with the research proposal to the corresponding author email: fernando.cardoso@embrapa.br.

## Ethics Statement

Ethical review and approval was not required for the animal study because it was developed using already existing datasets. Written informed consent was obtained from the owners for the participation of their animals in this study.

## Author Contributions

FC, OM, AD, NM, HB, MY, RP-W and BH conceived and designed the study. FC, OM, MY, RP-W, GC, and BH performed data analyses. FC wrote the manuscript draft. C-GG, NM and AM participated in the design and acquisition of data. All authors contributed to the article and approved the submitted version.

## Funding

Brazilian contributions and data funded by Conselho Nacional de Desenvolvimento Científico e Tecnológico, Grant/Award Number 305102/2018-4 and Empresa Brasileira de Pesquisa Agropecuária, Grant/Award Numbers 02.12.02.008.00, 02.13.10.002, and 12.13.14.014.00. Australian contributions and data were funded through Phases 2 and 3 of the Beef Cooperative Research Centre (http://www.beefcrc.com/). Australian contributions were funded by the Commonwealth Government funding through the CRC program, Meat and Livestock Australia and the Australian Centre for International Agricultural Research. The cattle used in the research were contributed by producers from the Northern Pastoral Group, and their financial support of this project is also gratefully acknowledged. This research was funded in part by the Bill & Melinda Gates Foundation and with UK aid from the UK Foreign, Commonwealth and Development Office (Grant Agreement OPP1127286) under the auspices of the Centre for Tropical Livestock Genetics and Health (CTLGH), established jointly by the University of Edinburgh, SRUC (Scotland’s Rural College), and the International Livestock Research Institute. The findings and conclusions contained within are those of the authors and do not necessarily reflect positions or policies of the Bill & Melinda Gates Foundation nor the UK Government. Work at The Roslin Institute was funded by Biotechnology and Biological Sciences Research Council through Institute Strategic Programme Grant funding (BBS/E/D/30002275). Nguni data collection and genotyping was supported through research grants from the Red Meat Research and Development of South Africa (RMRD-SA), Technology Innovation Agency (TIA) and the National Research Foundation (NRF) Grant No: CPRR14071676305.

## Conflict of Interest

The authors declare that the research was conducted in the absence of any commercial or financial relationships that could be construed as a potential conflict of interest.
